# Effectiveness of web-based personalised e‑Coaching lifestyle interventions

**DOI:** 10.1007/s12471-018-1200-7

**Published:** 2018-11-28

**Authors:** H. Yousuf, R. Reintjens, E. Slipszenko, S. Blok, G. A. Somsen, I. I. Tulevski, L. Hofstra

**Affiliations:** 1 Amsterdam UMC (Location: VU University Medical Center), Amsterdam, The Netherlands; 2 MyCLIC H4H BV, Amsterdam, The Netherlands; 3 Cardiologie Centra Nederland, Amsterdam, The Netherlands

**Keywords:** e-Coaching, e-Health, Lifestyle, Cardiovascular disease, Prevention, Lifestyle intervention

## Abstract

**Introduction:**

Interventions to reduce the impact of modifiable risk factors, such as hypercholesterolaemia, smoking, and overweight, have the potential to significantly decrease the cardiovascular disease burden. The majority of the global population is unaware of their own risk of developing cardiovascular disease. Parallel to the lack of awareness, a rise in obesity and diabetes is observed. e‑Health tools for lifestyle improvement have shown to be effective in changing unhealthy behaviour. In this study we report on the results of three different trials assessing the effectiveness of MyCLIC, an e‑Coaching lifestyle intervention tool.

**Methods:**

From 2008 to 2016 we conducted three trials: 1) HAPPY NL: a prospective cohort study in the Netherlands, 2) HAPPY AZM: a prospective cohort study with employees of Maastricht UMC+ and 3) HAPPY LONDON: a single-centre, randomised controlled trial with asymptomatic individuals who have a high 10-year CVD risk.

**Results:**

HAPPY NL and HAPPY AZM showed that e‑Coaching reduced cardiovascular risk. Both prospective trials showed a 20–25% relative reduction in 10-year cardiovascular disease risk. A lesser effect was seen in the HAPPY LONDON trial. A low frequency of logins suggests a low degree of content engagement in the e‑Coaching group, which could be age related as the mean age of the participants in the HAPPY LONDON study was high.

**Conclusion:**

e-Coaching using MyCLIC is a low cost and effective method to perform lifestyle interventions and has the potential to reduce the 10-year cardiovascular disease risk.

## What’s new?

- e-Coaching can be effective to reduce 10-year cardiovascular risk.
- The rise in smart phone use necessitates the transition to mobile health.
- Low engagement and high age are important limitations in the implementation of e‑Health.

## Introduction

### Background

Novel developments in technology affect almost every sector of healthcare and could play an important role in the reduction of the current epidemic of cardiovascular disease (CVD) [1]. By intervention of modifiable risk factors, such as hypercholesterolaemia, smoking, and overweight, the CVD burden can be significantly decreased [2, 3]. The majority of the global population is unaware of their risk of developing CVD, which goes hand in hand with the rise in obesity, and diabetes [4]. Moreover, the well-known InterHeart study demonstrated that 90% of all CVDs are related to unhealthy lifestyle [5].

e-Health tools for lifestyle improvement have shown to be effective in changing unhealthy behaviour. A personalised approach to electronic lifestyle coaching can improve lifestyle factors in individuals through identification of personal needs, setting personalised goals and using strategies to support change, in order to cause a sustainable lifestyle change [6]. Most lifestyle coaching tools have a single risk factor modification approach, such as cessation of smoking [7]. In our lifestyle intervention tool called MyCLIC (My Cardiac Lifestyle Intervention Coach) we chose a comprehensive approach in order to modify as many risk factors as possible.

Currently, most lifestyle interventions are performed according to the Dutch Collage of General Practitioners (NHG) guidelines in a face-to-face consultation by general practitioners (GPs) [8]. GPs have stated low confidence in their ability to impact the lifestyle of their patients. This goes together with a lack of consultation time and poor training in lifestyle intervention consultation. These findings suggest that a tool to support lifestyle coaching in the GPs office is needed [9].

### Aim

Here we report on the results of three consecutive trials, conducted from 2008 to 2016, to assess the clinical effectiveness of a personalised e‑Coaching lifestyle intervention to improve healthy lifestyle as a primary prevention tool in order to reduce the 10-year CVD risk score [10–12]. We performed three trials: 1) HAPPY NL: a prospective cohort study in the Netherlands campaigned via newspapers, 2) HAPPY AZM: a prospective cohort study as part of the ‘health week’ at Maastricht University Medical Center (formerly known as Academisch Ziekenhuis Maastricht (AZM)) with a relatively healthy population consisting of AZM employees, and 3) HAPPY LONDON: a single-centre, randomised controlled trial with asymptomatic individuals with high 10-year CVD risk.

### Methods and results

All participants gave written informed consent and all trials were approved by the national research ethics committee.

For all outcomes, normality was checked visually using histograms and q‑q plots. For normally distributed data, a t-test was used. For outcomes that were found to not be distributed normally, a Mann-Whitney U test was performed. A *p*-value of *p* *<* *0.05* was considered significant. All statistical analyses were performed using SPSS Statistics Version 25.0 (IBM Corp.).

### e-Coaching with MyCLIC

The e‑Coaching intervention, MyCLIC, has a duration of 1 year and begins and ends with a health check. With the data gathered from the health check and the online questionnaire, the heart risk and lifestyle score are calculated. The lifestyle score is calculated by a uniquely developed algorithm and shows how healthy the participant lives on a scale from 1–10, where 1 reflects a very unhealthy lifestyle and 10 a perfect lifestyle. This lifestyle algorithm takes several factors such as exercise, smoking, alcohol, nutrition, stress, and sleep into consideration. Subsequently, the results of the heart risk score, lifestyle score, and personalised advice are projected on a personalised website. The system is able to generate thousands of unique health plans, exactly meeting the demands of the individual patient or participant.

## HAPPY NL

### Study design

The HAPPY NL trial was a prospective cohort study that was campaigned via newspapers. In the campaign we invited newspaper readers to participate in our HAPPY Run preceded by a free health check. This resulted in inclusion and successful follow-up of 595 participants.

### Participants

During the health check we assessed height, weight, blood pressure, cholesterol, glucose, and lipid profile of the participants. All participants also filled out a lifestyle questionnaire. After 3 months of lifestyle e‑Coaching the participants were invited for a follow-up health check.

### Intervention

Two weeks after the first health check, the participants received their lifestyle score and heart risk results via email. During the following 3 months, the participants received lifestyle coaching via email.

### Results

The mean age of the 595 participants was 51 years and 295 were males. After the intervention, we observed a significant change in systolic blood pressure (138.6 vs 130.9 mm Hg, *p* < 0.001), diastolic blood pressure (81.5 vs 78.0 mm Hg, *p* < 0.001), weight (77.4 vs 76.1 kg, *p* < 0.001), BMI (26.0 vs 25.5, *p* < 0.001), glucose (5.6 vs 5.5 mmol/l, *p* < 0.001), total cholesterol (5.5 vs 5.3 mmol/l, *p* < 0.001), LDL-c (3.6 vs 3.5 mmol/l, *p* < 0.001), HDL-c (1.4 vs 1.3 mmol/l, *p* < 0.001), and triglycerides (1.1 vs 1.0 mmol/l, *p* < 0.001 (Tab. 1). Together, the reduction in CVD risk factors resulting from a better lifestyle resulted in a reduction of the PROCAM score (6.3 vs 5.5%, *p* < 0.001).

**Table 1:** Changes in cardiovascular risk parameters among participants of HAPPY NL

	test 1	test 2	*p*-value
weight (kg)	77.4	76.1	<0.001
BMI	26.0	25.5	<0.001
systolic blood pressure (mm Hg)	138.6	130.9	<0.001
diastolic blood pressure (mm Hg)	81.5	78.0	<0.001
glucose (mmol/l)	5.6	5.5	<0.001
cholesterol (total) (mmol/l)	5.5	5.3	<0.001
LDL cholesterol (mmol/l)	3.6	3.5	<0.001
HDL cholesterol (mmol/l)	1.4	1.3	<0.001
triglycerides (mmol/l)	1.1	1.0	<0.001
PROCAM (%)	6.3	5.5	<0.001

## HAPPY AZM

### Study design

The HAPPY AZM trial was a prospective cohort study performed in light of the AZM health awareness week. All employees at the AZM were invited to participate in HAPPY AZM; this resulted in inclusion and successful follow-up of 1,062 participants.

### Participants

Employees of the AZM were invited to fill out a lifestyle questionnaire. Exclusion was based on the following criteria: 1) not completing the lifestyle questionnaire, 2) having an established cardiovascular disease or 3) a condition that would prevent the participant from completing the trial. Included participants were invited to partake in a health check that we organised. After 1 year of lifestyle e‑Coaching the participants were invited for a second health check.

### Intervention

Two weeks after the first health check, the participants received the results of the health check by post. During 1 year, the participants received lifestyle advice via email and online in our e‑Coaching tool.

### Results

The mean age of the 1,062 participants was 44 years and 270 were males. After 1 year, we observed a significant change in systolic blood pressure (138.65 vs 136.55 mm Hg, *p* < 0.001), diastolic blood pressure (83.35 vs 82.14 mm Hg, *p* < 0.001), glucose (5.34 vs 5.47 mmol/l, *p* < 0.001), LDL-c (3.42 vs 3.28 mmol/l, *p* < 0.001), and HDL-c (1.40 vs 1.50 mmol/l, *p* < 0.001) (Tab. 2). As a result, the PROCAM score also dropped (2.62 vs 2.35%, *p* = 0.004). The intervention resulted in a net relative risk reduction of 22%.

**Table 2:** Changes in cardiovascular risk parameters among participants of HAPPY AZM

	test 1	test 2	*p*-value
weight (kg)	72.62	72.56	0.546
BMI	24.90	24.89	0.811
systolic blood pressure (mm Hg)	138.65	136.55	<0.001
diastolic blood pressure (mm Hg)	83.35	82.14	<0.001
Total cholesterol (mmol/l)	5.35	5.31	0.064
HDL cholesterol (mmol/l)	1.40	1.50	<0.001
LDL cholesterol (mmol/l)	3.42	3.28	<0.001
triglycerides (mmol/l)	1.20	1.21	0.536
glucose (mmol/l)	5.34	5.47	<0.001
PROCAM (%)	2.62	2.35	0.004

## HAPPY LONDON

### Study design

The HAPPY LONDON trial was a two-arm, non-blinded, randomised controlled trial in a single-centre setting. During 18 months, 402 adults with a high 10-year CVD risk were included in our trial. All participants underwent a 6-month follow-up.

### Participants

Inclusion was based on: 1) age between 40–74 years, 2) a 10-year CVD risk score of ≥10%, 3) access to Internet, and 4) adequate understanding of the English language. Exclusion was based on: 1) having an established cardiovascular disease or 2) a condition that would prevent the participant from completing the trial. Participant recruitment was done by postal invitation. Assessment of lifestyle took place via MyCLIC. During health check visits at the William Harvey Heart Centre assessment of weight, height, BMI, blood pressure, cholesterol (HDL, LDL, total), triglycerides, and glucose took place. Participants were randomised to receive e‑Coaching or not, on top of face-to-face lifestyle consultation, which is the standard of care (SOC) [13]. Three follow-up visits were planned, after 2 weeks, 3 and 6 months.

### Intervention

Participants in the e‑Coaching group received 6 months of tailored advice and were able to enter their MyCLIC pages with personal logins.

### Results

The mean age of the 402 participants was 65.5 years and 270 were males. Greater improvements in systolic blood pressure (mean change −3.18 vs −1.688), diastolic lipid profile (mean change −0.42 vs −0.763), and weight (mean change −1.22 vs −0.763) (Tab. 3) were observed in the e‑Coaching group; however, statistical significance was not reached due to the large variation in outcomes. Improvements in lifestyle factors, although not significant, were mostly seen in participants of the e‑Coaching group. These participants were exercising more, consuming more vegetables, and generally had a better lifestyle score.

**Table 3:** Final results and mean-change in cardiovascular risk parameters among participants of HAPPY LONDON

	e-Coaching group	mean change	SOC group	mean change	*p*-value
weight (kg)	78.49	−1.22	79	−0.763	0.1
BMI	27.4	−0.42	27.1	−0.247	0.07
systolic blood pressure (mm Hg)	129.5	−3.18	130.7	−1.688	0.27
diastolic blood pressure (mm Hg)	76.7	−2.37	78	−2.076	0.67
total cholesterol (mmol/l)	4.8	−0.16	4.9	−0.197	0.6
HDL cholesterol (mmol/l)	1.6	−0.03	1.6	−0.017	0.64
LDL cholesterol (mmol/l)	2.6	−0.1	2.8	−0.142	0.56
triglycerides (mmol/l)	1.2	−0.08	1.2	−0.113	0.56
glucose (mmol/l)	5.6	−0.29	5.5	−0.266	0.77
QRISK2 (%)	19.2	0.14	18.9	0.01	0.63
framingham (%)	16.1	−1.23	16.6	−1.37	0.79

## Discussion

HAPPY NL and HAPPY AZM showed that e‑Coaching does reduce cardiovascular risk. Both trials showed a 20–25% relative reduction in 10-year CVD risk. This reduction of relative risk in developing CVDs in healthy individuals is similar to the relative risk reduction that is caused by medical treatment of CVD risk factors, such as medication to treat hypertension and hypercholesterolaemia [14, 15].

In the HAPPY NL trial, we also investigated differences in the effects of e‑Coaching on different socioeconomic groups and observed no differences in the impact of intervention. This suggests that MyCLIC is suitable for widespread implementation in a variety of social classes and educational levels.

The intervention in the HAPPY LONDON trial showed less effect on the 10-year CVD risk. Although favourable trends in exercise, blood pressure and BMI were shown, statistical significance was not reached.

Several reasons may explain the lack of statistical difference, such as large inter-individual variation in the items investigated and underpowering of the study. In addition, a low frequency of logins suggests a low degree of content engagement in the e‑Coaching group, which could be age related as the mean age of the participants in the HAPPY LONDON study was high, compared to the other two trials (mean age: 65.5 years vs 51 years and 44 years). In addition, a SOC lifestyle intervention was done in all participants, and performed by a physician researcher trained to provide lifestyle advice. This may have led to added positive impact on the SOC, and less extractable outcome of the e‑Coaching. Hence, we could only assess the added impact of e‑Coaching to high quality SOC and not as a replacement of SOC.

## Coping with the expected increase in prevalence of cardiovascular disease

GPs play a key role in the management and detection of unhealthy lifestyle behaviour [16]. It is projected that the burden of cardiovascular disease in the Netherlands will increase by 50% within a 25-year span, due to ageing and unhealthy lifestyle behaviour [17]. This will put further pressure on the limited time and recourses for intervention in the GPs practice [18]. Due to the rapidly growing epidemic of CVDs and the rise in cardiovascular risk management (CVRM), a great need for the implementation of e‑Coaching is in sight. MyCLIC may not only be an effective tool for lifestyle interventions, but could also assist health professionals in the assessment of individual healthcare needs [19].

Health insurance companies are recognising the benefit of intervening at an early stage, which resulted in the reimbursement of preventive strategies to combat diabetes mellitus (DM) [20]. This led to a major shift towards prevention of DM from the hospital towards primary healthcare. CVRM is prone to undergo a similar shift, which together with an efficient and cost-effective e‑Health tool, will enable the primary care professionals to respond adequately to the CVD epidemic [21].

## Successful e‑Coaching tool: the next stage

An effective lifestyle intervention e‑Coaching tool must precisely identify lifestyle issues, personal needs of the user, and provide adequate coaching to cause sustainable lifestyle change [22]. MyCLIC is an algorithm based e‑Coaching tool which provides the user with a personalised lifestyle coach through data driven decision making, and cardiovascular risk profiling of the user, which is in concordance with the Dutch CVRM guidelines [23]. The mechanism of personalised intervention selection could be further optimised with the use of artificial intelligence [24].

It remains a challenge to achieve high engagement with online content in e‑Health tools, which was apparent in the HAPPY LONDON trial. [25] Nevertheless, several strategies can be applied to achieve higher engagement. We designed engagement strategies in collaboration with GP practice groups and patients. Achievements, which have been shown to be effective, can consist of app notifications based on milestones in health growth, such as days of smoking cessation [26]. In addition, micro-learning is implemented to create an interactive coaching method. In micro-learning short snippets of information are provided, and alternated with short questions. These strategies result in greater effectiveness of e‑Coaching, due to activating intrinsic motivation and supporting self-efficacy of the user [27].

**Fig. 1:**
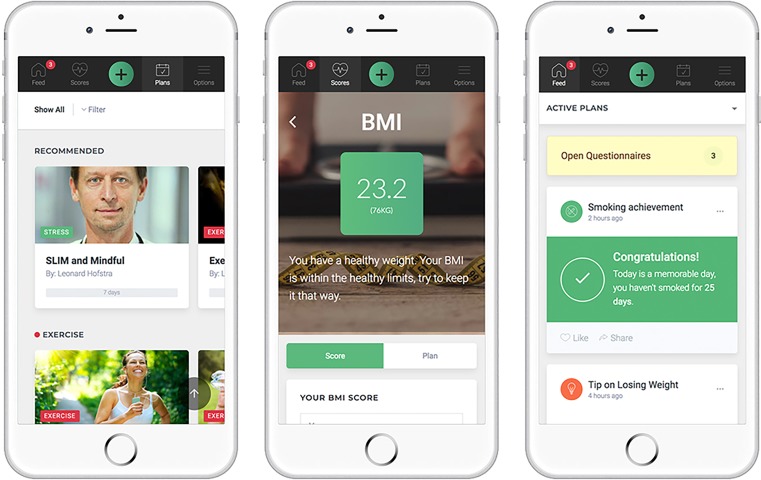
An impression of the MyCLIC native mobile application. From left to right: (1) an overview of personalised health plans, (2) overview of a risk factor (BMI), and (3) the participants personal home screen

Recently, smart phones have been embraced by a rapidly increasing number of people and have become a large part of our lives. This trend is expected to grow in coming years, and creates a great opportunity for health tools to be accessible anywhere at any time. However, high age could be a limitation in the use smart phones. Nevertheless, the explosive rise in smart phone use could result in the shift from e‑Health to mobile Health (m-Health) and necessitates a redesign of health tools into m‑Health tools [28]. Based on these trends, we have decided to build a MyCLIC native app (Fig. 1).

## Conclusion

e-Coaching with MyCLIC is an appropriate method to perform effective lifestyle interventions. Intervention with our e‑Coaching tool has been shown to cause a 20–25% relative risk reduction of the 10-year CVD risk. Widespread implementation of MyCLIC could have great socioeconomic impact, as it has great potential in the prevention of cardiovascular disease. However, high engagement of the user with the e‑Coaching tool is needed to achieve significant lifestyle change, in which high age could be limiting factor. In addition, the rapid adoption of smart phones and their extensive use requires adaptation of the program to a mobile version.
